# Anidulafungin compared with fluconazole in severely ill patients with candidemia and other forms of invasive candidiasis: Support for the 2009 IDSA treatment guidelines for candidiasis

**DOI:** 10.1186/cc10514

**Published:** 2011-10-25

**Authors:** Daniel H Kett, Andrew F Shorr, Annette C Reboli, Arlene L Reisman, Pinaki Biswas, Haran T Schlamm

**Affiliations:** 1 The Miller School of Medicine at the University of Miami, Jackson Memorial Hospital, 1611 NW 12th Avenue, Miami, FL 33136, USA; 2 Pulmonary Critical Care, Washington Hospital Center, 110 Irving Street, Washington, DC 20010, USA; 3 Faculty Affairs, Cooper Medical School of Rowan University, Ferry Terminal Building, Two Aquarium Drive, Camden, NJ 08103, USA; 4 Specialty Business Unit, Pfizer Pharmaceuticals, 235 East 42nd Street, New York, NY 10017-5703, USA; 5 Specialty Care Business Unit, Pfizer Pharmaceuticals, 500 Arcola Rd, Collegeville, PA 19426-3982, USA

## Abstract

**Introduction:**

During the past decade, the incidence of *Candida *infections in hospitalized patients has increased, with fluconazole being the most commonly prescribed systemic antifungal agent for these infections. However, the 2009 Infectious Diseases Society of America (IDSA) candidiasis guidelines recommend an echinocandin for the treatment of candidemia/invasive candidiasis in patients who are considered to be "moderately severe or severely" ill. To validate these guidelines, clinical trial data were reviewed.

**Methods:**

A secondary analysis of data from a previously published prospective, randomized, double-blind clinical trial was performed; it compared anidulafungin with fluconazole for the treatment of invasive candidiasis and candidemia. Patients with critical illness were identified at study entry by using the following criteria: Acute Physiology and Chronic Health Evaluation (APACHE) II score of ≥ 15, evidence of severe sepsis (sepsis and one or more end-organ dysfunctions) present, and/or patient was in intensive care. Global response rates were compared at the end of intravenous study treatment (the primary end point of the original study) and all-cause mortality at 14 and 28 days from study entry in this group.

**Results:**

The patients (163 (66.5%) of 245) fulfilled at least one criterion for critical illness (anidulafungin, *n *= 89; fluconazole, *n *= 74). No significant differences were found in baseline characteristics between the two treatment groups. The global response rate was 70.8% for anidulafungin and 54.1% for fluconazole (*P *= 0.03; 95% confidence interval (CI): 2.0 to 31.5); all-cause mortality was 10.1% versus 20.3% at 14 days (*P *= 0.08; 95% CI, -0.9 to 21.3) and was 20.2% versus 24.3% at 28 days (*P *= 0.57; 95% CI, -8.8 to 17.0) for anidulafungin and fluconazole, respectively.

**Conclusions:**

In this *post hoc *analysis, anidulafungin was more effective than fluconazole for treatment of severely ill patients with candidemia, thus supporting the 2009 IDSA guidelines.

**Trial registration:**

Clinicaltrials.gov NCT00058682.

## Introduction

The incidence of *Candida *infections in hospitalized patients has increased over the past decade [1-6]. *Candida *spp. are now the third most frequent cause of nosocomial bloodstream infections in the intensive care unit (ICU), accounting for up to 10% to 15% of all septicemia cases in this setting [1,4-6]. In the United States, the incidence of *Candida *bloodstream infections in surgical ICUs was reported to be 9.82 per 1,000 admissions [7], and, in a large international survey, the prevalence was 6.9 per 1,000 ICU admissions [8]. *Candida *bloodstream infections in the ICU are commonly associated with high mortality [1,6,9-11], and a European study showed mortality in ICU patients with candidemia to be significantly higher than that in the overall population of hospitalized patients [12]. Worldwide, *C. albicans *remains the most common *Candida *spp. responsible for candidemia, whereas non-*albicans *spp. compose 40%; however, geographic and age-related differences in the causative *Candida *spp. have been noted [13-16].

Fluconazole remains the most commonly prescribed systemic antifungal agent for treatment of candidemia and other forms of invasive candidiasis [8,10,17]. However, the 2009 Infectious Diseases Society of America (IDSA) treatment guidelines favor an echinocandin (anidulafungin, caspofungin, or micafungin) as initial therapy for candidemia in patients with "moderately severe to severe" illness, with fluconazole reserved for patients who are less severely ill [18]. The echinocandins are highly active against a broad spectrum of *Candida *spp. (anidulafungin minimum inhibitory concentration (MIC)_50_/MIC_90 _0.06/2 μg/ml; caspofungin MIC_50_/MIC_90_, 0.03/0.25 μg/ml, and micafungin MIC_50_/MIC_90 _0.015/1 μg/ml), including *C. glabrata *and *C. krusei*, against which the azole antifungal agents are known to have less activity [19]. In prospective, randomized clinical trials, caspofungin has been demonstrated to be at least as effective as amphotericin B [20], and micafungin was shown to be as effective as liposomal amphotericin B [21] and caspofungin [22]. Only one study to date has compared an echinocandin with fluconazole for the treatment of candidemia [23]. In that prospective, randomized, double-blind study, global success at end of intravenous treatment was 75.6% in the anidulafungin group, compared with 60.2% in the fluconazole group (95% confidence interval (CI) 3.9 to 27.0) [23]. The results were similar for other efficacy end points. Based on a review of the distribution of baseline Acute Physiology and Chronic Health Evaluation (APACHE) II scores in this study [23], we suspected that a substantial proportion of patients would be considered severely ill at study entry. We saw this as an opportunity to compare outcomes in these patients, allowing us to validate the 2009 IDSA candidemia treatment guidelines.

## Materials and methods

In the previously published study, 245 adult patients with culture-confirmed candidemia were randomly assigned to receive initial study treatment with either intravenous (IV) anidulafungin or fluconazole. Patients were administered IV anidulafungin (200 mg on day 1, and then 100 mg daily) or IV fluconazole (800 mg on day 1, and then 400 mg daily) for ≥ 10 days. Therapy with IV study drug was optionally followed by oral fluconazole if certain predefined conditions were met. The study protocol was approved by the institutional review board at each of the participating centers, and all patients, or their legal representatives, provided written informed consent before enrollment. The trial was registered on Clinicaltrials.gov (NCT00058682). Complete details on study design and results were published elsewhere [23].

Patients in this study were considered to be severely ill if they met any of the following criteria at study entry: APACHE II score ≥ 15 (which was above the median APACHE II score of patients in the primary study [23] and is associated with a mortality of 20% or greater in patients with severe sepsis [24]); requirement for intensive care; or evidence of severe sepsis present. The original study required that patients have a diagnosis of candidemia and meet at least two of the criteria for systemic inflammatory response syndrome (SIRS) [25]. Consensus definitions for end-organ dysfunction were developed based on the clinical and laboratory data collected during the study: *cardiovascular dysfunction *was defined as a need for a vasopressor agent within 24 hours of study entry; *renal dysfunction *was defined as a calculated creatinine clearance < 30 ml/min [26] or need for dialysis at study entry; *respiratory dysfunction *was defined as a requirement for mechanical ventilation; *hepatic dysfunction *was defined as total bilirubin level greater than twice the upper limit of normal (ULN) or alanine aminotransferase (ALT), aspartate aminotransferase (AST), or alkaline phosphatase greater than 5 times ULN; *multiple organ dysfunction *was defined as the presence of more than one single-organ dysfunction.

The primary analysis was a comparison of the investigator-assessed global (that is, combined clinical and microbiologic) response at the end of IV study treatment (referred to hereafter as end of treatment) with either anidulafungin or fluconazole. Secondary analyses included comparison of global response in each of the three subpopulations: (a) patients with APACHE II score ≥ 15; (b) patients in an ICU; and (c) patients with severe sepsis. To identify any potential imbalances between treatment groups, baseline demographic and clinical characteristics were compared by using the Fisher Exact test or *t *test, as appropriate. The limited sample size did not provide sufficient power to adjust for possible confounders or multiple comparisons within groups. All-cause mortality was compared at 14 days (the median duration of treatment with anidulafungin) and at 28 days, by using the Fisher Exact test. Survival duration at 14 and 28 days was also compared by using Kaplan-Meier analysis and the log-rank test.

## Results

**Figure 1:**
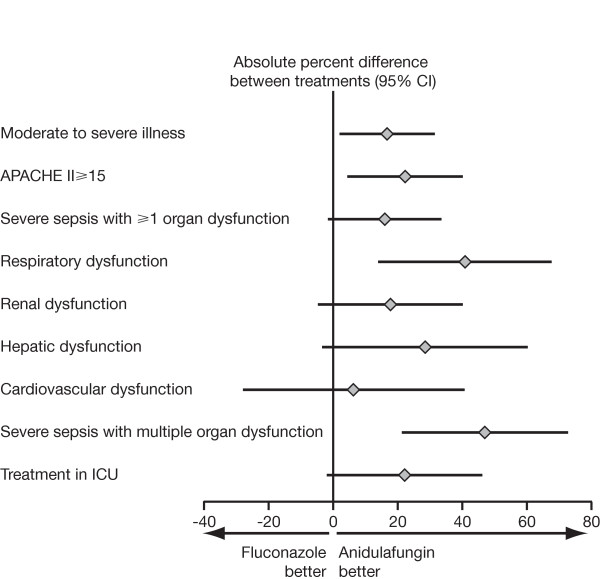
**Difference in global response at end of treatment among severely ill patients and the various subpopulations**.

**Figure 2:**
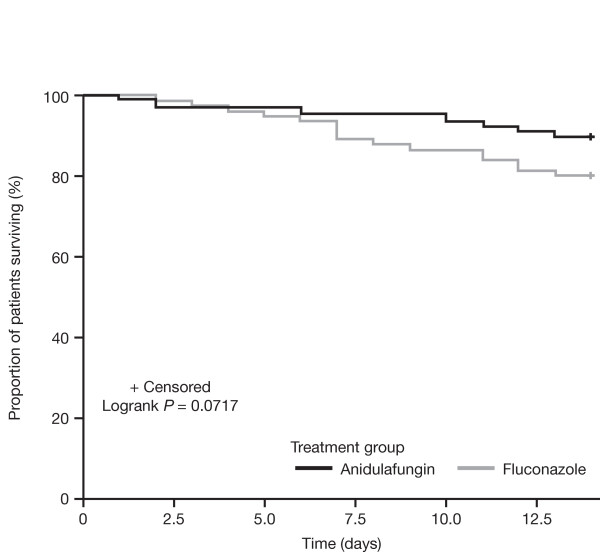
**Kaplan-Meier analysis of survival to 14 days among severely ill patients with candidemia**.

Of the 245 patients, 163 (66.5%) fulfilled at least one of the criteria for critical illness: 89 received anidulafungin, and 74 received fluconazole. We found no significant differences in baseline characteristics between the two treatment groups (Table 1). In patients with critical illness overall, the global response rate was 70.8% for anidulafungin and 54.1% for fluconazole (*P *= 0.03; 95% CI, 2.0 to 31.5); all-cause mortality at 14 days was 10.1% versus 20.3% (*P *= 0.08 by the Fisher Exact test; 95% CI, -0.9 to 21.3), and at 28 days was 20.2% versus 24.3% (*P *= 0.57 by the Fisher Exact test; 95% CI, -8.8 to 17.0) for anidulafungin and fluconazole, respectively (Table 2 and Figure 1). The Kaplan-Meier comparison of survival at day 14 is presented in Figure 2 (*P *= 0.07 by log-rank test).

**Table 1:** Baseline demographic and clinical characteristics in severely ill patients

	Anidulafungin (*n *= 89)	Fluconazole (*n *= 74)
Age (years), mean (SD)	60.2 (16.4)	62.2 (16.4)
APACHE II score, mean (SD)	18.0 (7.0)	17.3 (6.8)
Gender, *n *(%)		
Male	45 (50.6)	41 (55.4)
Female	44 (49.4)	33 (44.6)
Race, *n *(%)		
White	65 (73.0)	59 (79.7)
Black	19 (21.3)	9 (12.2)
Other	5 (5.6)	6 (8.1)
Absolute neutrophil count, *n *(%)		
**≤ **500	3 (3.4)	4 (5.4)
> 500	86 (96.6)	70 (94.6)
Risk factors for candidemia, *n *(%)		
Central venous catheter	71 (79.8)	58 (78.4)
Broad-spectrum antibiotics	65 (73.0)	53 (71.6)
Recent surgery	37 (41.6)	34 (45.9)
Recent hyperalimentation (TPN)	25 (28.1)	16 (21.6)
Malignancy	23 (25.8)	16 (21.6)
Immunosuppressive therapy	14 (15.7)	19 (25.7)
Transplant	5 (5.6)	4 (5.4)
Source of isolates, *n *(%)		
Candidemia	85 (95.5)	69 (93.2)
Intra-abdominal infection	4 (4.5)	5 (6.8)
*Candida *spp., *n *(%)		
Single isolate	81 (91.0)	69 (93.2)
*C. albicans*	45 (50.6)	43 (58.1)
*C. glabrata*	15 (16.9)	14 (18.9)
*C. parapsilosis*	10 (11.2)	4 (5.4)
*C. tropicalis*	9 (10.1)	6 (8.1)
Mixed	8 (9.0)	5 (6.8)

APACHE, Acute Physiology and Chronic Health Evaluation; SD, standard deviation; TPN, total parenteral nutrition.

**Table 2:** Global response at end of treatment among severely ill patients and the various subpopulations

Patient group	Global response at end of treatment				
		**Anidulafungin**	**Fluconazole**	***P *value**	**Absolute difference (95% CI)**
				
		***n *(%)**			

Moderate to severe illness	Success	63/89 (70.8)	40/74 (54.1)	0.034	16.7% (2.0, 31.5)
	Failure	26/89 (29.2)	34/74 (45.9)		
APACHE II **≥ **15	Success	43/63 (68.3)	23/50 (46.0)	0.022	22.3% (4.3, 40.2)
	Failure	20/63 (31.7)	27/50 (54.0)		
Severe sepsis with **≥ **1 organ dysfunction	Success	42/62 (67.7)	29/56 (51.8)	0.092	16.0% (-1.6, 33.5)
	Failure	20/62 (32.3)	27/56 (48.2)		
Respiratory dysfunction	Success	16/22 (72.7)	7/22 (31.8)	0.015	40.9% (14.0, 67.8)
	Failure	6/22 (27.3)	15/22 (68.2)		
Renal dysfunction	Success	26/36 (72.2)	18/33 (54.5)	0.142	17.7% (-4.7, 40.1)
	Failure	10/36 (27.8)	15/33 (45.5)		
Hepatic dysfunction	Success	13/18 (72.2)	7/16 (43.8)	0.163	28.5% (-3.4, 60.4)
	Failure	5/18 (27.8)	9/16 (56.3)		
Cardiovascular dysfunction	Success	6/13 (46.2)	8/20 (40.0)	1.000	6.2% (-28, 40.7)
	Failure	7/13 (53.8)	12/20 (60.0)		
Severe sepsis with multiple organ dysfunction	Success	16/21 (76.2)	7/24 (29.2)	0.003	47.0% (21.3, 72.8)
	Failure	5/21 (23.8)	17/24 (70.8)		
Treatment in ICU	Success	24/35 (68.6)	13/28 (46.4)	0.122	22.1% (-1.9, 46.2)
	Failure	11/35 (31.4)	15/28 (53.6)		

### APACHE II score ≥ 15

Of the original 245 patients, 113 (46.1%) had an APACHE II score ≥ 15 at study entry: 63 were treated with anidulafungin, and 50 with fluconazole. In this group, global response at the end of treatment was significantly higher with anidulafungin than with fluconazole (68.3% versus 46.0%; *P *< 0.05; 95% CI, 4.3 to 40.2) (Table 2 and Figure 1). All-cause mortality at 14 days was 14.3% for anidulafungin and 28.0% for fluconazole, with a difference of 13.7 percentage points (95% CI, -1.4 to 28.9; *P *= 0.10 by the Fisher Exact test). All-cause mortality at 28 days was 27.0% for anidulafungin and 34.0% for fluconazole, with a difference of 7.0 percentage points (95% CI, -10.0 to 24.1; *P *= 0.54 by the Fisher Exact test). No statistically significant differences were found in the comparisons of Kaplan-Meier estimates of survival at day 14 (*P *= 0.07 by log-rank test) or day 28 (*P *= 0.42 by log-rank test).

### Severe sepsis

Of the original 245 patients, 118 (48.2%) met the criteria for severe sepsis (SIRS with end-organ dysfunction) at study entry: 62 were treated with anidulafungin, and 56 with fluconazole. Global response at end of treatment (Table 2 Figure 1) was 67.7% for anidulafungin and 51.8% for fluconazole (*P *= 0.092; 95% CI, -1.6 to 33.5). All-cause mortality at 14 days was 12.9% for anidulafungin and 19.6% for fluconazole, with a difference of 6.7 percentage points (95% CI, -6.6 to 20.1; *P *= 0.45 by Fisher Exact test); all-cause mortality at 28 days was 25.8% for anidulafungin and 25.0% for fluconazole, with a difference of -0.8 percentage points (95% CI, -17.0 to 14.9; *P *= 0.93 by Fisher Exact test). No statistically significant differences were noted in the comparisons of Kaplan-Meier estimates of survival at day 14 (*P *= 0.33 by log-rank test) or day 28 (*P *= 0.98 by log-rank test).

Of the 118 patients with severe sepsis, 44 patients had respiratory dysfunction, 69 patients had renal dysfunction, 34 patients had hepatic dysfunction, 33 patients had cardiovascular dysfunction, and 45 had multiple end-organ dysfunction. The global responses in each of these subpopulations are presented in Table 2 and Figure 1. For patients with multiple end-organ dysfunction, global response at end of treatment (Table 2 and Figure 1) was 76.2% for anidulafungin and 29.2% for fluconazole (*P *< 0.05; 95% CI, 21.3 to 72.8). In this subpopulation, all-cause mortality at 14 days was 19.0% for anidulafungin and 29.2% for fluconazole, with a difference of 10.1 percentage points (95% CI, -15.0 to 34.9; *P *= 0.50 by Fisher Exact test), and all-cause mortality at 28 days was 38.1% for anidulafungin and 33.3% for fluconazole, with a difference of -4.8 percentage points (95% CI, -33.0 to 23.3; *P *= 0.77 by Fisher Exact test). Again, no statistically significant differences were seen in the comparisons of Kaplan-Meier estimates of survival at day 14 (*P *= 0.45 by log-rank test) or day 28 (*P *= 0.85 by log-rank test).

### ICU patients

Hospital records for 166 of the 245 study patients were reviewed to identify patients in an ICU at study entry. Of the 166 patients, 63 were found to be in an ICU at study entry, and of these, 35 patients received anidulafungin and 28 received fluconazole. Global response at end of treatment (Table 2 and Figure 1) was 68.6% for anidulafungin and 46.4% for fluconazole (*P *= 0.122; 95% CI, -1.9 to 46.2). All-cause mortality at 14 days was 8.6% for anidulafungin and 21.4% for fluconazole (95% CI, -4.9 to 30.7; *P *= 0.17 by Fisher Exact test). All-cause mortality at 28 days was 17.1% for anidulafungin and 28.6% for fluconazole (95% CI, -9.4 to 32.3; *P *= 0.36 by Fisher Exact test). No statistically significant differences were found in the comparisons of Kaplan-Meier estimates of survival at day 14 (*P *= 0.16 by log-rank test) or day 28 (*P *= 0.27 by log-rank test).

### Safety

The number of treatment-related adverse events was similar in the two treatment arms: 44 treatment-related adverse events were reported in 22 (23.7%) of 93 patients receiving anidulafungin, and 40 treatment-related adverse events were reported in 21 (27.3%) of 77 patients receiving fluconazole. All-causality adverse events leading to discontinuation of the study drug occurred in 13 (14%) of 93 patients receiving anidulafungin and in 18 (23.4%) of 77 patients receiving fluconazole (*P *= 0.16). The majority of these treatment-limiting adverse events were signs and symptoms of worsening disease rather than adverse events related to medication toxicity.

## Discussion

The 2009 IDSA candidemia treatment guidelines recommend initial treatment with an echinocandin in patients with "moderately severe to severe" illness [18]. To validate these guidelines for the treatment of candidemia, we identified a group of patients with critical illness at study entry in a prospective, randomized clinical trial that compared anidulafungin with fluconazole [23]. In this group, global response at end of treatment was significantly higher with anidulafungin than with fluconazole.

We believe that this global-response observation supports the IDSA candidemia guidelines, as well as current European guidelines that recommend an echinocandin for treatment of candidemia in hemodynamically unstable ICU patients [27]. Three recent reports, however, were unable to confirm the advantage of an echinocandin over other systemic antifungal agents for the treatment of candidemia in ICU patients. Two *post hoc *subgroup analyses of data collected in prospective randomized studies reported similar clinical responses and all-cause mortality in ICU patients receiving initial treatment with an echinocandin (micafungin or caspofungin) when compared with amphotericin B-based comparators [28,29]. Similarly, a *post hoc *analysis of patients with candidemia requiring mechanical ventilation failed to demonstrate a survival advantage for an echinocandin compared with fluconazole, even after adjusting for nontreatment factors [30]. In our analysis, similar patients with candidemia and respiratory failure who were treated with anidulafungin had better global responses at the end of treatment compared with those with fluconazole, but no significant difference in survival was found. In contrast to other studies, we did not limit our analysis to ICU patients. To be more consistent with the IDSA guidelines, we identified patients who were considered to be "moderately severe to severely ill" and included patients outside of an ICU setting.

The recent IDSA clinical practice guidelines suggest that more severely ill patients with candidemia should receive echinocandins as first-line therapy [18]; however, they do not provide a definition for "moderately severe to severe" illness. To test these recommendations, we had to create a clinically valid definition for this patient population. We first attempted to identify patients who were likely to have been severely ill because they were in an ICU at study entry. However, this information was not collected in the casebook, and hospital medical records were available for review for only two thirds of the study population. In addition, criteria for ICU admission were potentially different across study sites. We then considered all patients with a baseline APACHE II score ≥ 15 at study entry to be severely ill, because this score is associated with a mortality of 20% or greater in septic patients [24]. However, these APACHE II scores were calculated at study entry, and not necessarily at the time of ICU admission, where these scores have been validated. We then identified all patients with sepsis and evidence of organ dysfunction at study entry because these patients would also be considered to be severely ill. To accomplish this, we generated and applied consensus definitions for severe sepsis with respiratory, renal, hepatic, and/or cardiovascular dysfunction based on the information collected during the study. The data required to categorize other types of end-organ (for example, central nervous system) dysfunction, or those with septic shock, however, were not collected in a consistent manner in the study case-report form.

We considered patients who met any of these three criteria to be severely ill for the purpose of this analysis. This strategy has the advantage of allowing us to identify these patients independent of hospital ward location, as well as APACHE II scores at study entry. However, prospective studies using more-formal definitions for severely ill patients with candidemia should be considered. In addition, step-down therapy to fluconazole should be evaluated for patients who have improved clinically after initial therapy with an echinocandin and who are infected with an organism that is likely to be fluconazole susceptible, in line with IDSA guidelines [18]. As reported by Reboli *et al. *[23], the majority of pathogens isolated in this study, including *C. glabrata *isolates, were susceptible to fluconazole. Of note, several critically ill patients with *C. parapsilosis *infection had a satisfactory response to anidulafungin.

Our study has important limitations. It is a *post hoc *analysis of the prospective, randomized, double-blind, clinical trial that compared anidulafungin with fluconazole for the treatment of candidemia [23]. The definitions used to classify patients as severely ill relied on data obtained from case-report forms and from a *post hoc *chart review encompassing the majority of randomized patients. Additionally, a substantial overlap existed between these definitions; therefore, some patients were included in more than one of the resulting three groups. Although a numerically greater global response was noted with anidulafungin versus fluconazole in essentially all subsets of severely ill patients, classification of patients into three groups resulted in relatively small sample sizes, and for several of these groups, the difference in global response rates did not approach statistical significance. In addition, subdivision into small sample sizes meant that this analysis was underpowered to detect differences in survival between the two treatment groups, and no statistically significant differences were observed. In the anidulafungin and fluconazole comparative trial [23], a trend toward improved survival was seen in patients receiving anidulafungin (*P *= 0.10 when comparing Kaplan-Meier estimates of survival).

In summary, in our analysis of severely ill patients with candidemia, anidulafungin was associated with improved global response at end of study treatment compared with fluconazole. Although derived from a *post hoc *analysis of clinical trial data, these observations support the 2009 IDSA treatment guidelines, which recommend an echinocandin for primary treatment of moderately severe to severely ill patients with candidemia. Additional studies should be conducted the better to define the critical care patient population(s) that would most benefit from initial treatment with an echinocandin.

## Key messages

• Fluconazole is the most common systemic antifungal agent prescribed for treatment of candidemia and invasive candidiasis; however, the 2009 Infectious Diseases Society of America (IDSA) treatment guidelines favor an echinocandin as initial therapy for candidemia in patients with moderately severe to severe illness.

• This retrospective review of data from the prospective, randomized, double-blind clinical trial demonstrated that anidulafungin is more effective than fluconazole for treatment of severely ill patients with candidemia, thus supporting the 2009 IDSA guidelines.

## Abbreviations

APACHE: Acute Physiology and Chronic Health Evaluation; ALT: alanine aminotransferase; AST: aspartate aminotransferase; CI: confidence interval; ICU: intensive care unit; IDSA: Infectious Diseases Society of America; IV: intravenous; MIC: minimum inhibitory concentration; SD: standard deviation; SIRS: systemic inflammatory response syndrome; TPN: total parenteral nutrition; ULN: upper limit of normal.

## Competing interests

DHK has received research support from Akers Bioscience, Astellas, Biogel/Hibiclens, Ortho-McNeil, and Pfizer; has been a consultant to Astellas, Lilly, and Pfizer; and has served as a speaker for Astellas, Cubist, Glaxo, Lilly, Ortho-McNeil, Pfizer, and Wyeth. AFS has served as a consultant to, speaker for, and/or has received research support from Astellas, Merck, and Pfizer. ACR has received clinical research grant support from Merck and Pfizer, has been a consultant for Merck and Pfizer, and has been a lecturer for Pfizer. ALR, PB, and HTS are employees of Pfizer Inc.

## Authors' contributions

DK, HS, PB, and AR contributed to the design of the study. All authors contributed to the acquisition of data, analysis, and interpretation of data. PB designed and analyzed the statistical analysis of the data presented in this article. All authors were involved in revising the draft manuscript. All authors read and approved the final manuscript.
